# Supplementary material for: Acid-tolerant injectable bioadhesive for sutureless repair of large gastric perforation
Source: Nat Commun. 2026 Mar 24;17:4364. doi: 10.1038/s41467-026-71031-9 (PMC13172332; doi:10.1038/s41467-026-71031-9)
Supplement: Supplementary file 1 — Supplementary Information [file 41467_2026_71031_MOESM1_ESM.pdf]

Supplementary information

Acid-tolerant injectable bioadhesive for sutureless repair of large gastric perforation

Ze Wang[1,2,3,#], Bo Cao[1,3,#], Longsong Li[4,#], Hao Cui[1], Bo Wei[1,3]*, Jianxin Cui[1,3]*, Xing Wang[2,5]*

[1]Department of General Surgery, the First Medical Center, Chinese PLA General Hospital, Beijing 100853, China.

[2]Beijing National Laboratory for Molecular Sciences, Institute of Chemistry, Chinese Academy of Sciences, Beijing 100190, China.

[3]Medical School of Chinese PLA, Beijing 100853, China.

[4]Department of Gastroenterology, the First Medical Center, Chinese PLA General Hospital, Beijing 100853, China.

[5]University of Chinese Academy of Sciences, Beijing 100049, China.

[#]These authors contributed equally to this work.

*Correspondence:

E-mail: weibo@301hospital.com.cn (B.W.); cuijx_doctor@163.com (J.C.); wangxing@iccas.ac.cn (X.W.)

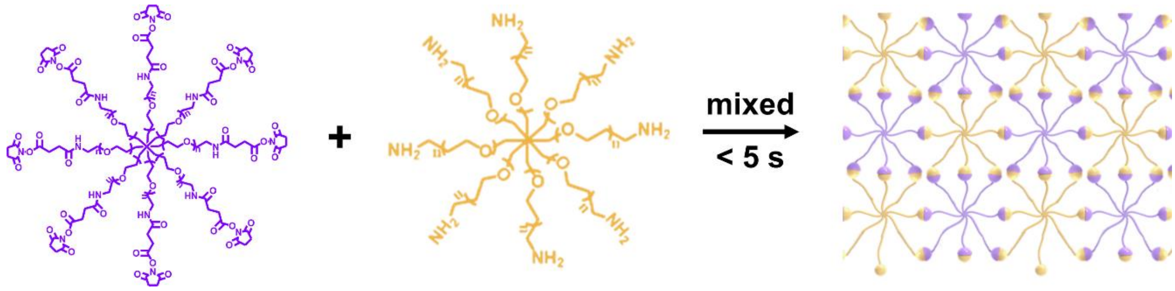

Supplementary Figure 1. Schematic preparation of OSSA hydrogel by the mixture of Octa-PEG-SSA and Octa-PEG-NH_2 solution within 5 s.

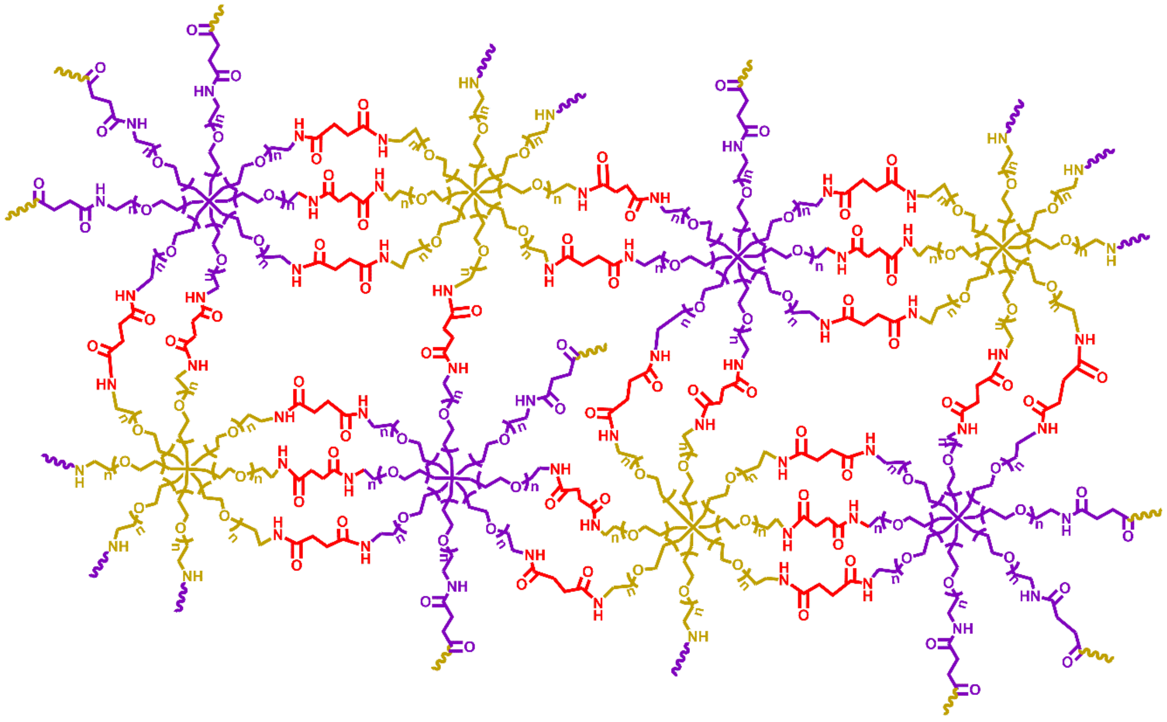

Supplementary Figure 2. Schematic diagram of amide-linked crosslinking network.

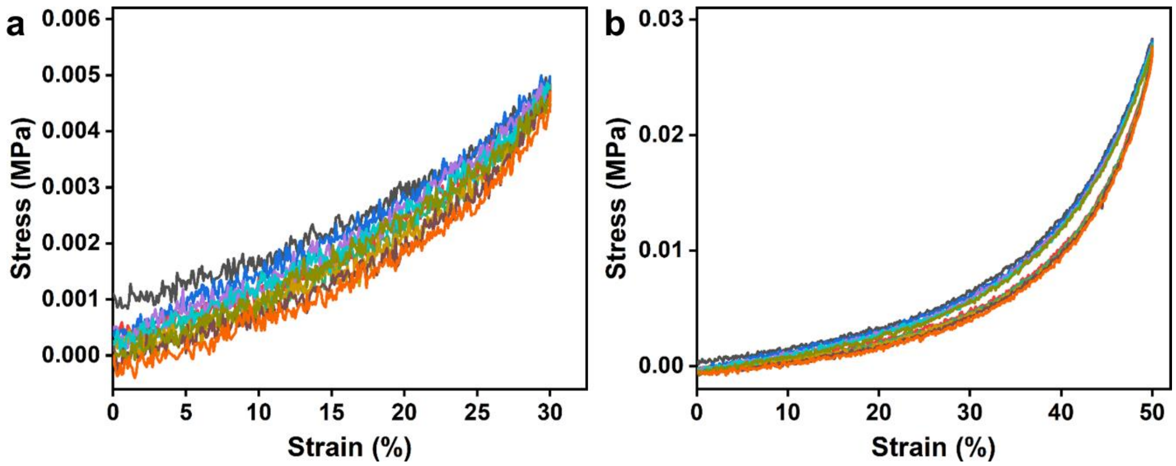

Supplementary Figure 3. Cyclic stress-strain compressive curves at a strain of **a**) 30% and **b**) 50% of the OSSA hydrogel. Source data are provided as a Source Data file.

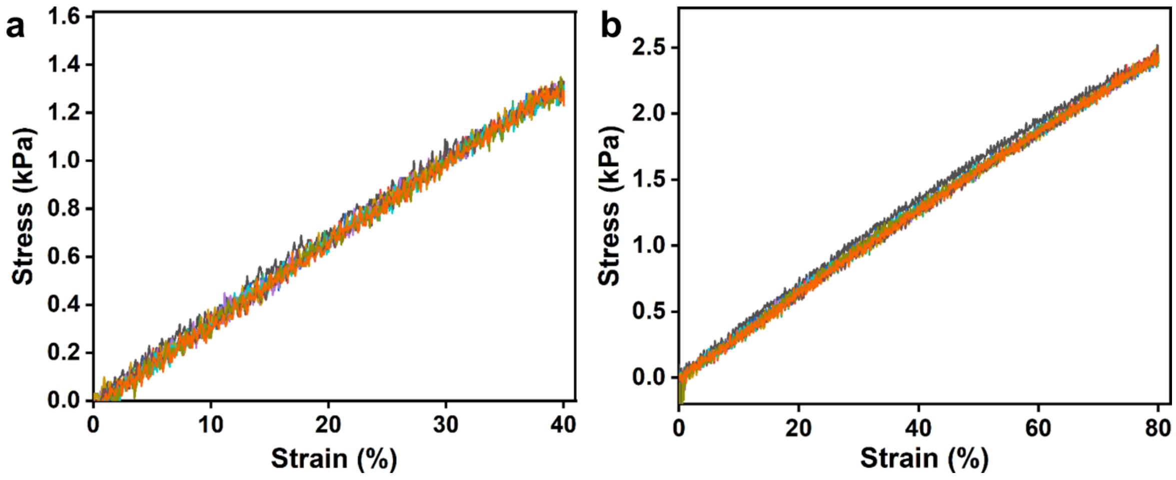

Supplementary Figure 4. Cyclic stress-strain tensile curves at a strain of **a**) 40% and **b**) 80% of the OSSA hydrogel. Source data are provided as a Source Data file.

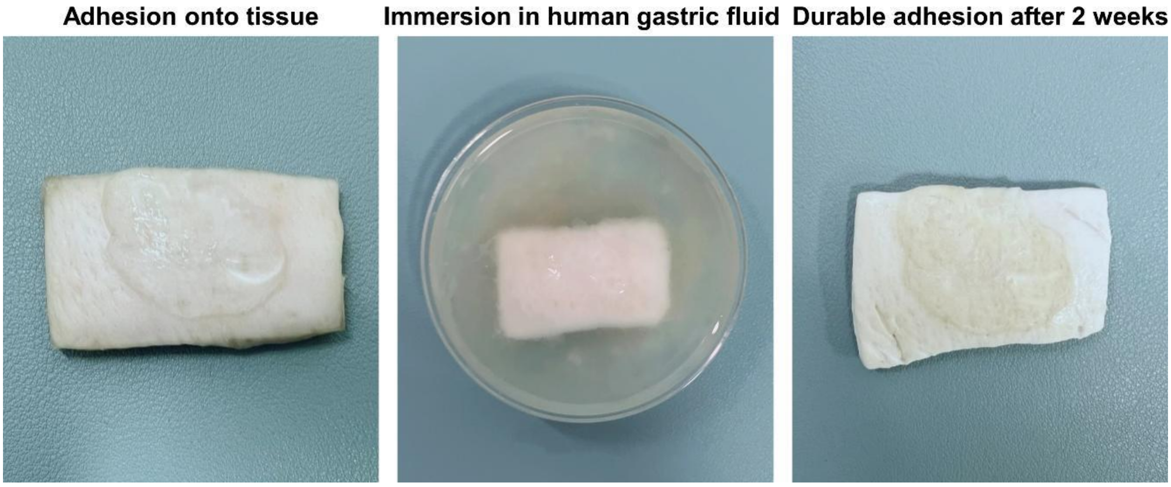

Supplementary Figure 5. Representative images of OSSA hydrogel adhering onto the porcine skin immersed in human gastric fluid for 2 weeks.

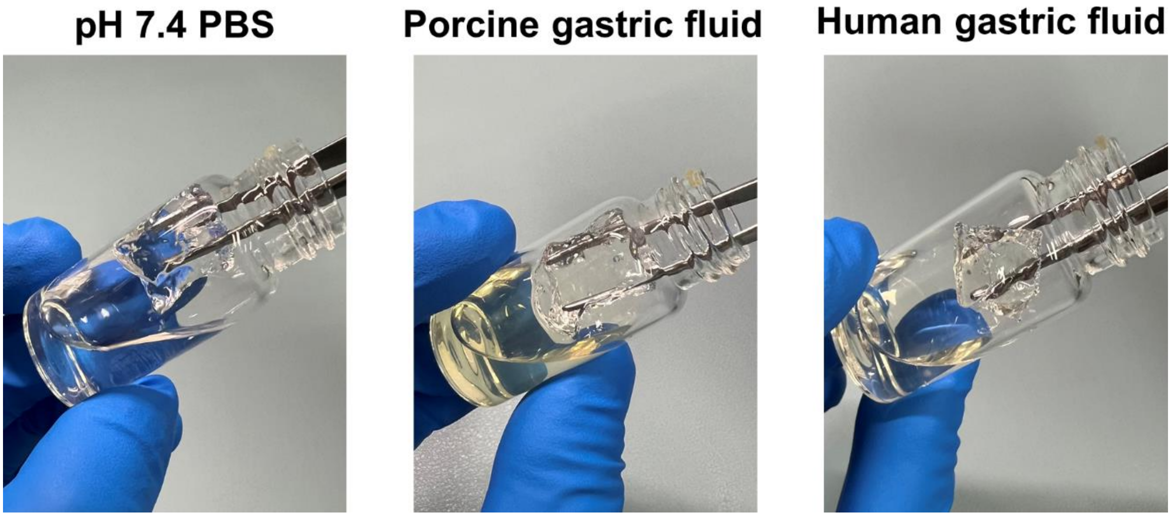

Supplementary Figure 6. Representative images of tolerance of OSSA hydrogel against pH 7.4 PBS, porcine gastric juice, and human gastric juice.

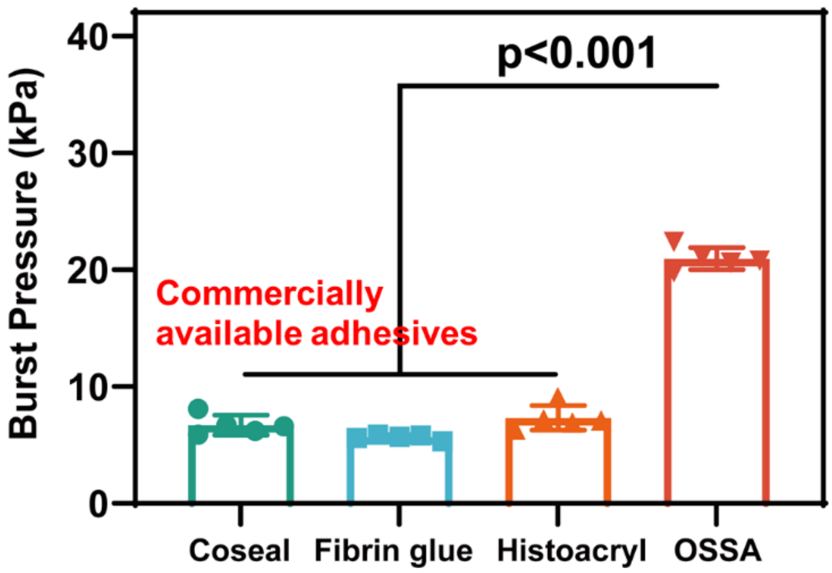

Supplementary Figure 7. Burst pressure of *ex vivo* porcine colons with a 4-mm-diameter defect sealed by the OSSA and commercially available tissue adhesives (Coseal, Fibrin glue, and Histoacryl). Data are presented as mean ± SEM (n=5 independent samples). P values were determined by one-way ANOVA with Least Significant Difference post hoc test. Source data are provided as a Source Data file.

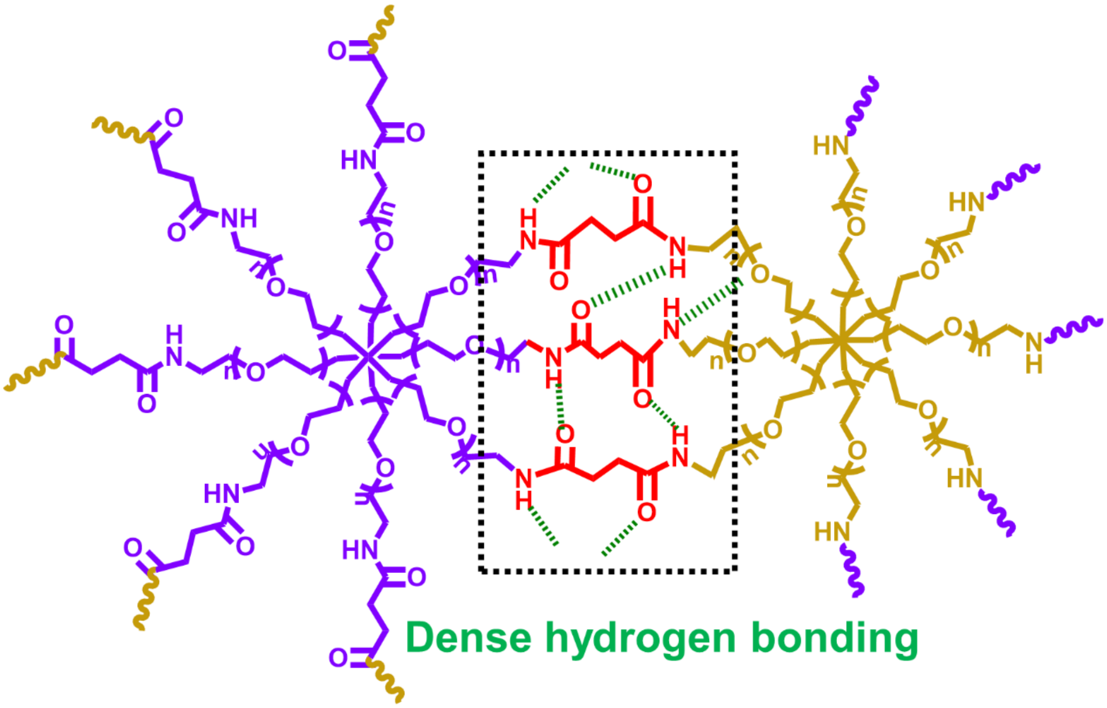

Supplementary Figure 8. Schematic diagram of an intermediate structure of OSSA hydrogel with crosslinking junction of amide bonds and dense hydrogen bonding domains.

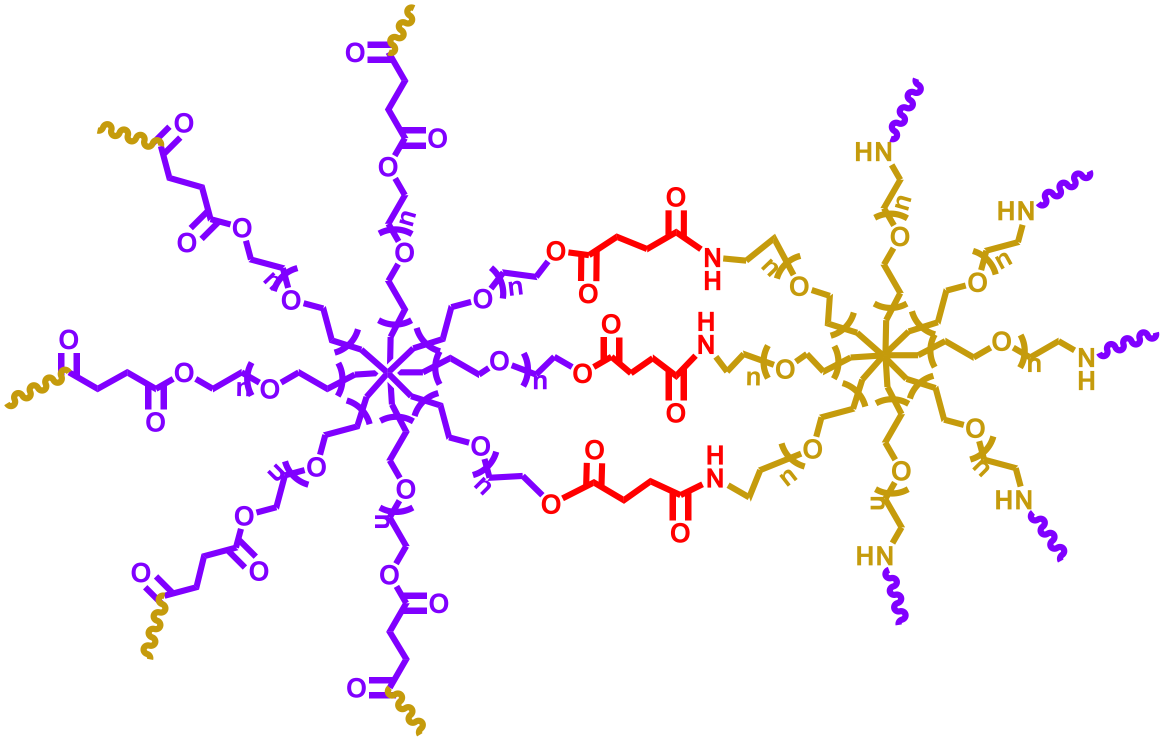

Supplementary Figure 9. Schematic diagram of an intermediate structure of OSS hydrogel with crosslinking junction of ester and amide bonds.

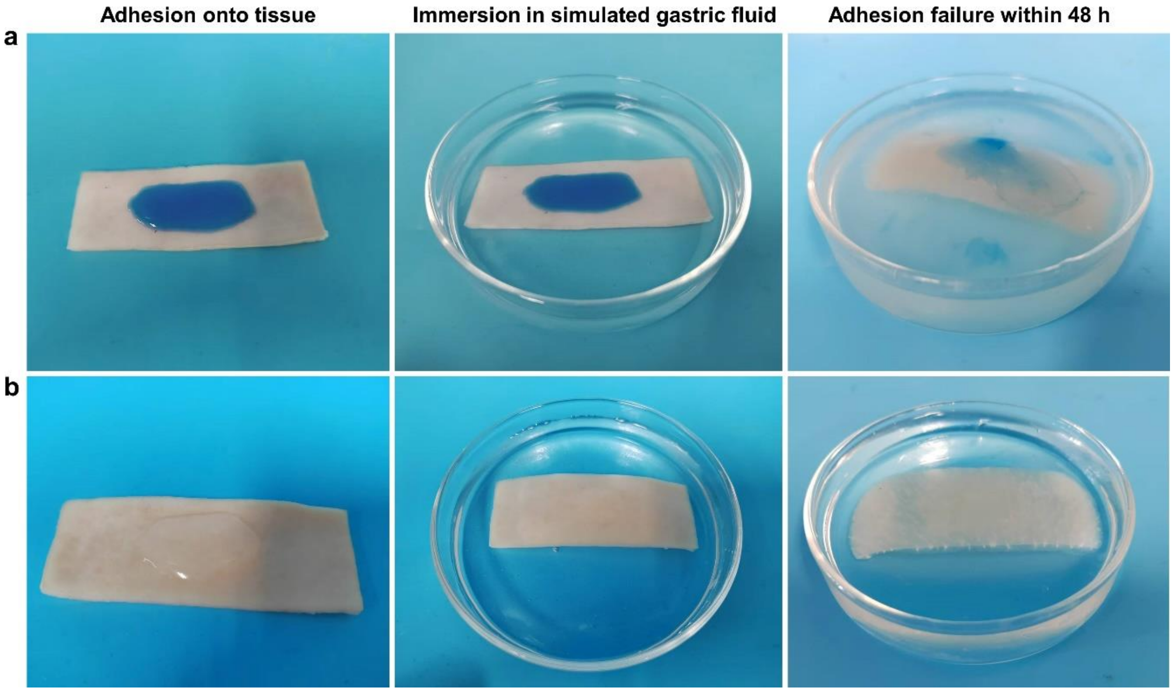

Supplementary Figure 10. Representative images of **a**) OSS hydrogel and **b**) Coseal sealant adhering onto the porcine skin immersed in simulated gastric fluids for 2 days.

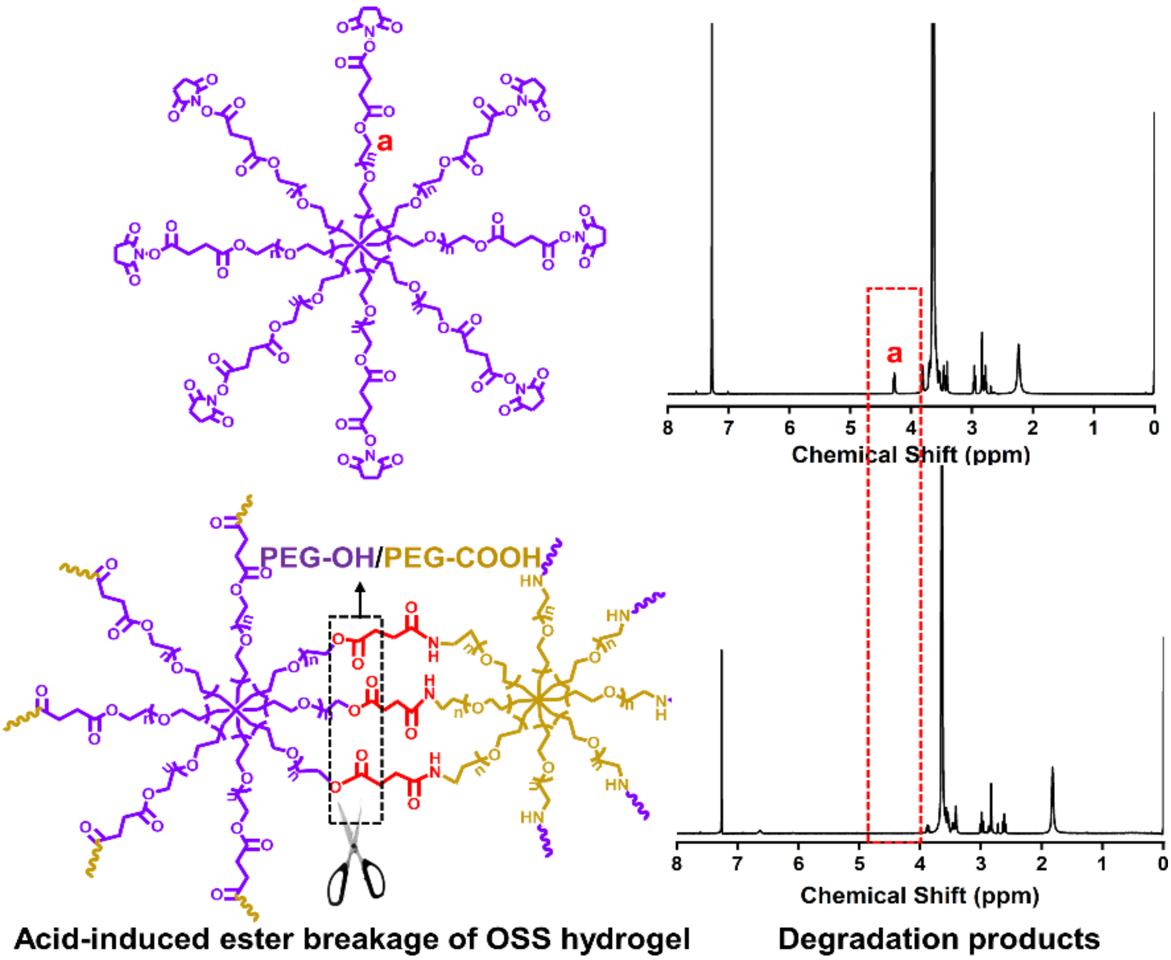

Supplementary Figure 11. ^{1}H NMR spectra of Octa-PEG-SS-succinate polymer and degradation products of OSS hydrogel after incubation in acidic conditions for 2 days.

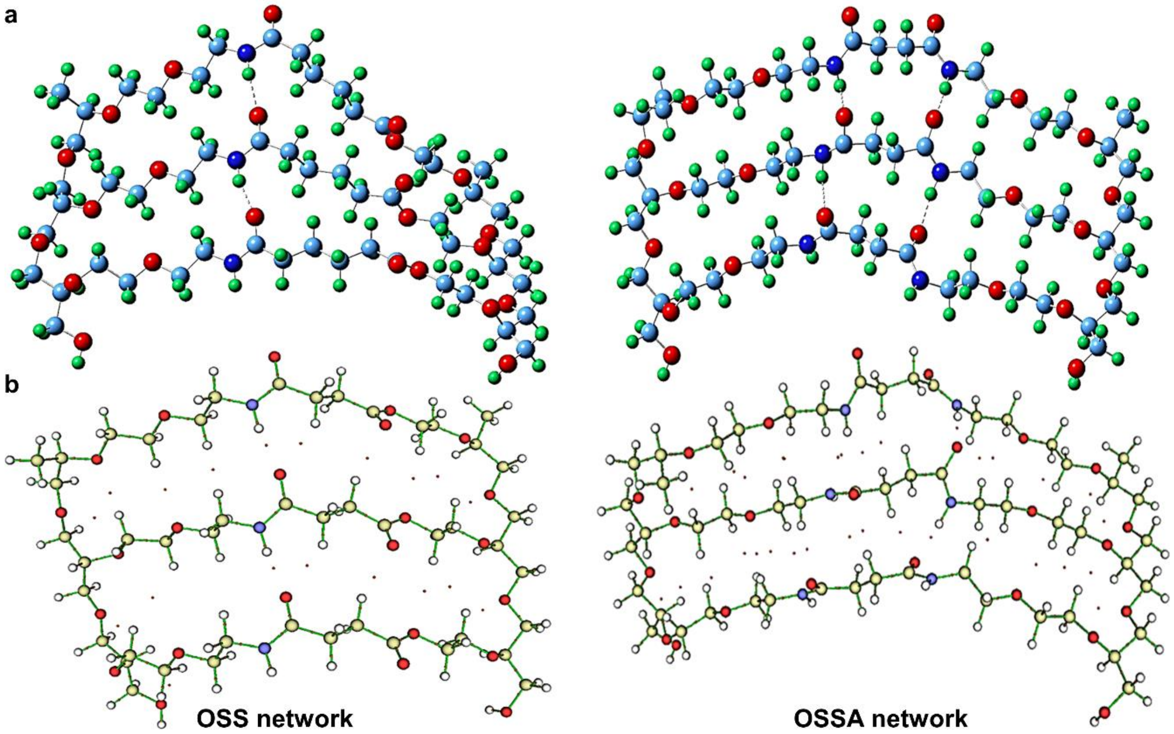

Supplementary Figure 12. **a**) Schematic diagram illustrating N-H···O hydrogen bond interaction within the OSS network and OSSA network. **b**) BCPs of the OSS network and OSSA network.

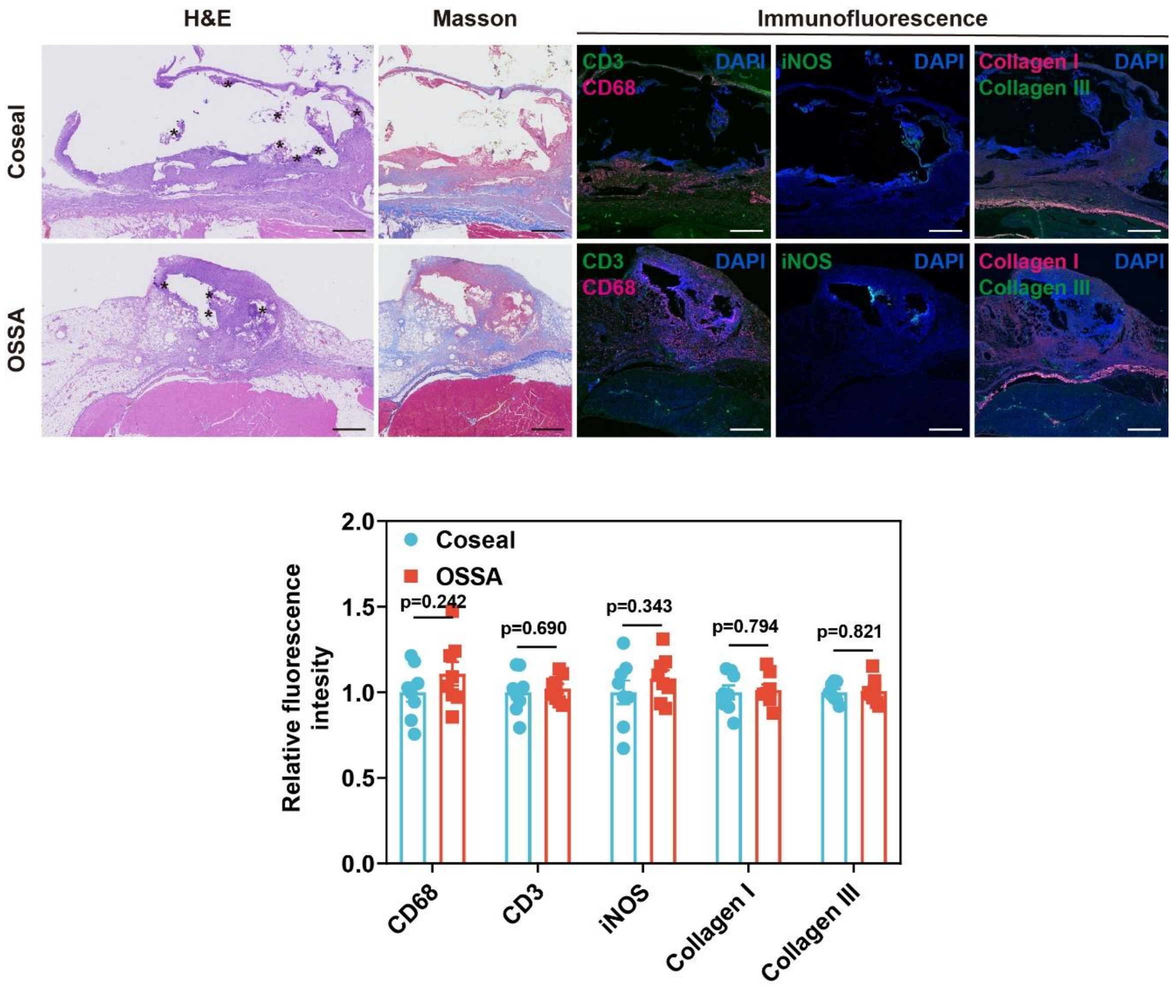

Supplementary Figure 13. The representative images of H&E, Masson staining, and immunofluorescence of the tissues marked with CD68, CD3, iNOS, Collagen I, and Collagen III for Coseal and OSSA groups at Day 3. Scale bar: 500 µm. The histogram below shows the relative immunofluorescent densities of immunofluorescence. Data are presented as mean ± SEM (n=8 independent samples). Three areas per tissues were randomly selected and analyzed. P values were determined by two-sided Student's *t* test. Source data are provided as a Source Data file.

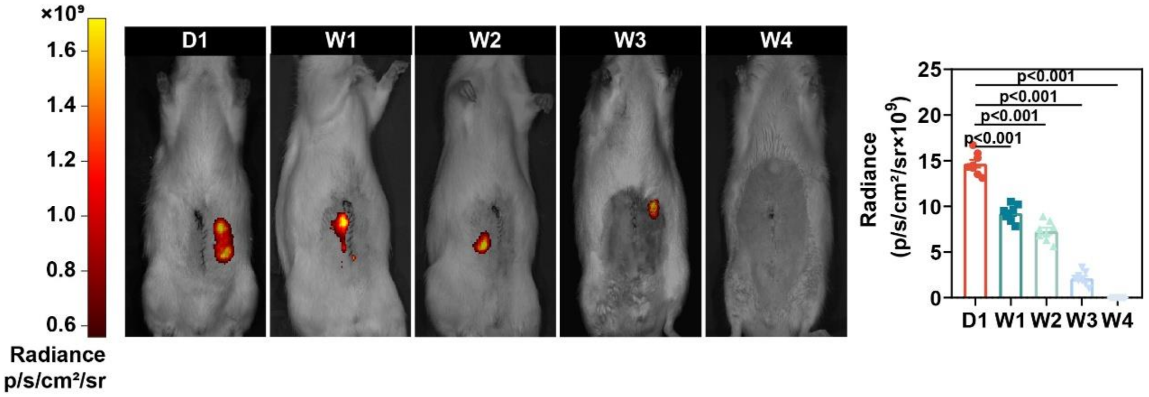

Supplementary Figure 14. The bioluminescent images (left) and the histogram (right) showing the remaining OSSA hydrogel implanted on omentum majus at Day 1, Week 1, 2, 3, 4, respectively. Data are presented as mean ± SEM (n=8 independent samples). P values were determined by one-way ANOVA with Least Significant Difference post hoc test. Source data are provided as a Source Data file.

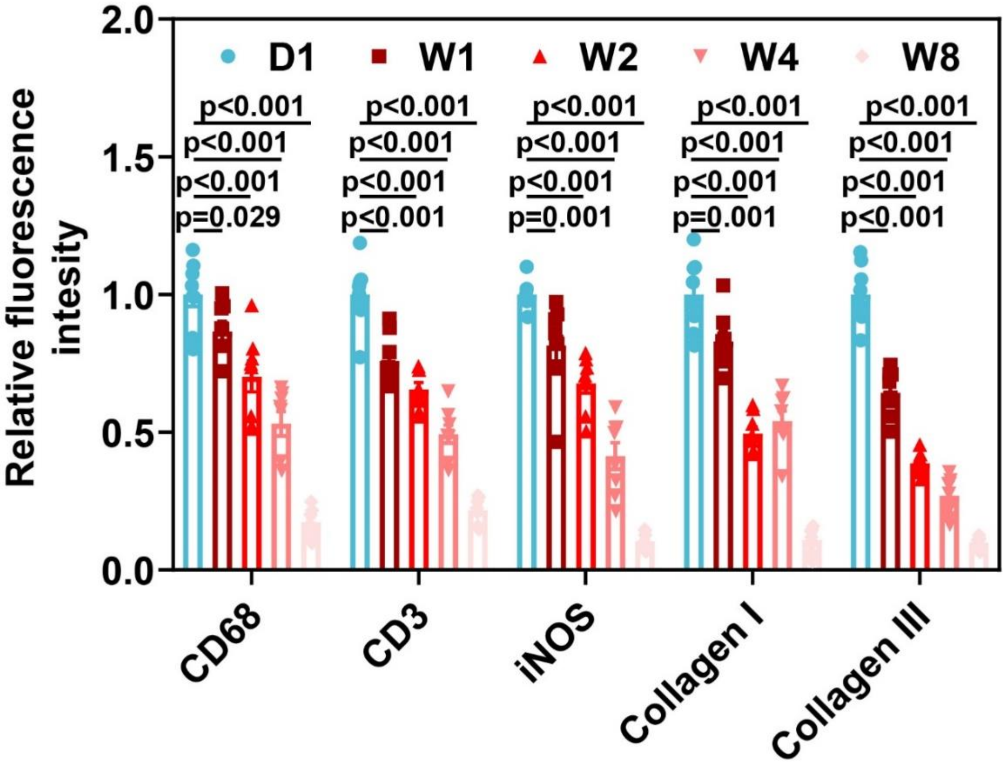

Supplementary Figure 15. The relative immunofluorescent densities of tissues marked with CD68, CD3, iNOS, Collagen I, and Collagen III after subcutaneous implantation with the OSSA hydrogel at Day 1, Week 1, 2, 4, 8, respectively. Data are presented as mean ± SEM (n=8 independent samples). P values were determined by one-way ANOVA with Least Significant Difference post hoc test. Source data are provided as a Source Data file.

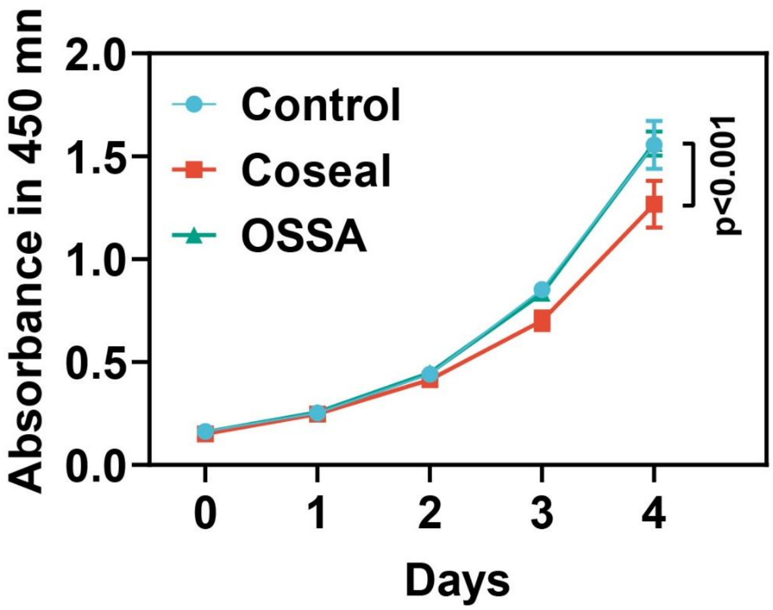

Supplementary Figure 16. CCK-8 assay of the viability of GES-1 cells treated with the control (DMEM), Coseal, and OSSA hydrogel extracts at Day 0, 1, 2, 3, 4, respectively. Data are presented as mean ± SEM (n=3 independent samples). Three contemporary measurements from the same experimental batch were performed for average. P values were determined by two-way ANOVA followed by Least Significant Difference post hoc test. Source data are provided as a Source Data file.

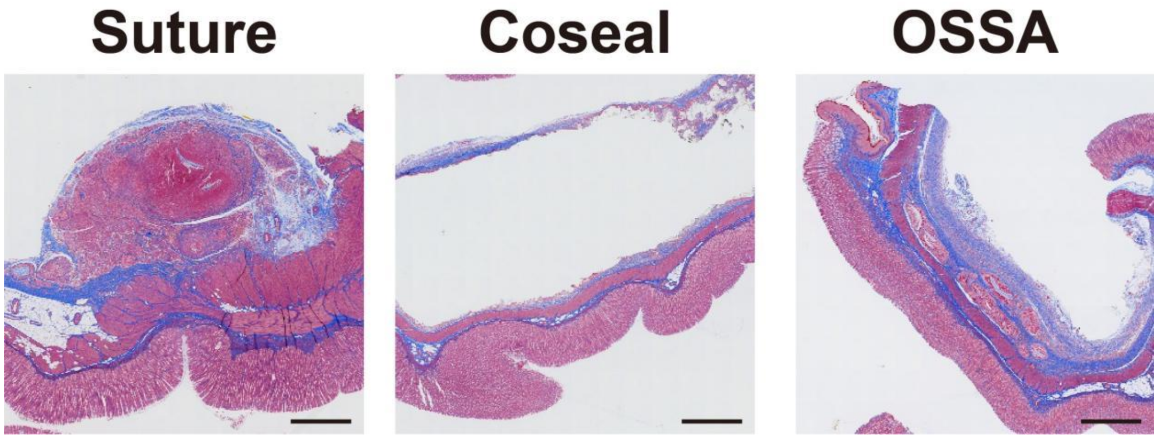

Supplementary Figure 17. Representative Masson staining for stomach sealed by the suture, Coseal, and OSSA bioadhesives after 4 weeks, respectively (n=8 independent samples). Three areas per tissues were randomly selected and analyzed.

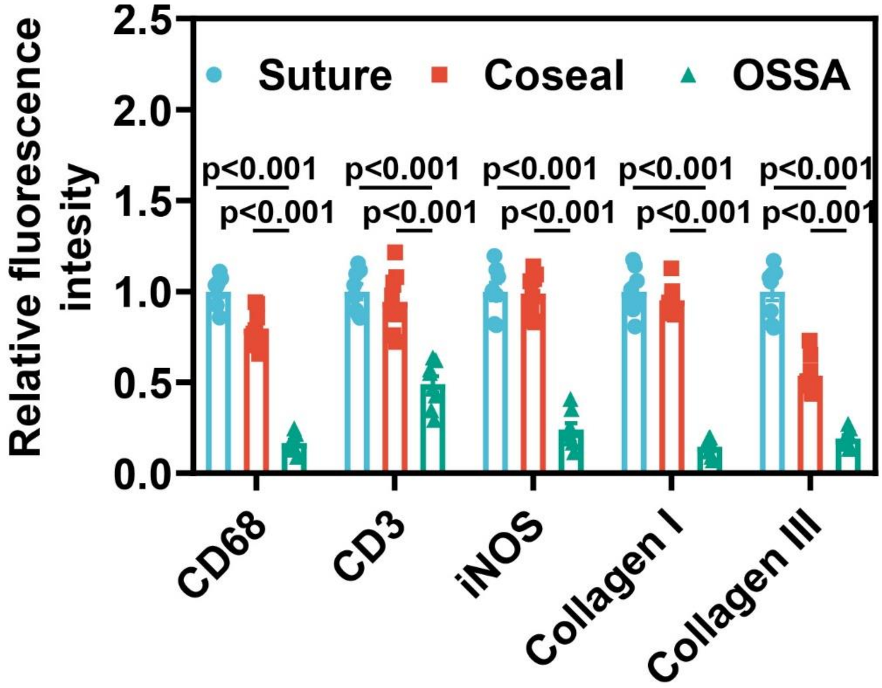

Supplementary Figure 18. The relative immunofluorescent densities of gastric tissues marked with CD68, CD3, iNOS, Collagen I, and Collagen III after sealing with the suture, Coseal, and OSSA bioadhesives after 4 weeks, respectively. Data are presented as mean ± SEM (n=8 independent samples). P values were determined by one-way ANOVA with Least Significant Difference post hoc test. Source data are provided as a Source Data file.

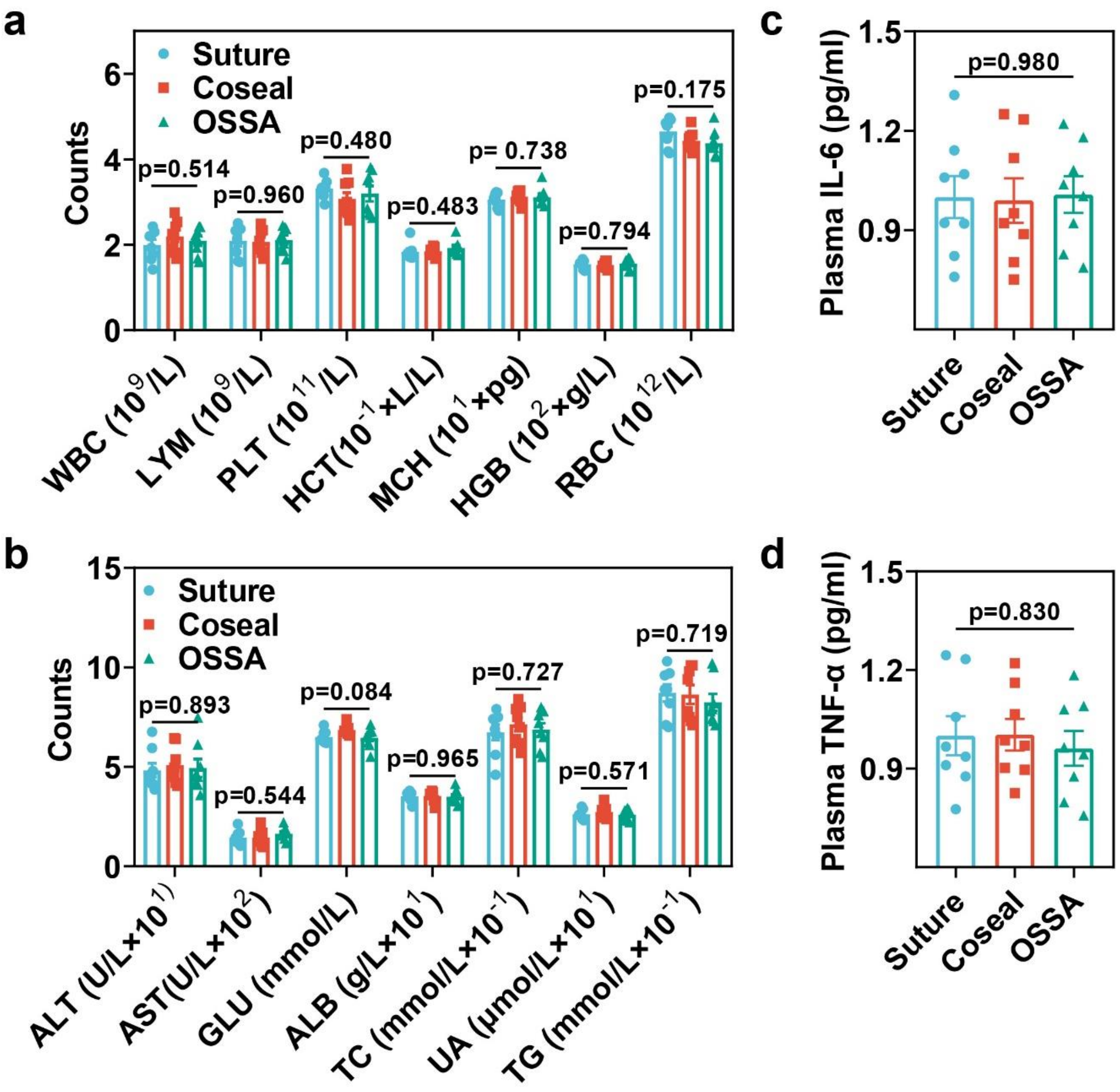

Supplementary Figure 19. ***In vivo*** **biocompatibility of the OSSA bioadhesive using rat models. a-d**) Blood routine, biochemistry, IL-6, and TNF-α of rats with gastric defects treated with the suture, Coseal, and OSSA bioadhesives, respectively. Data are presented as mean ± SEM (n=8 independent samples). P values were determined by one-way ANOVA with Least Significant Difference post hoc test. Source data are provided as a Source Data file.

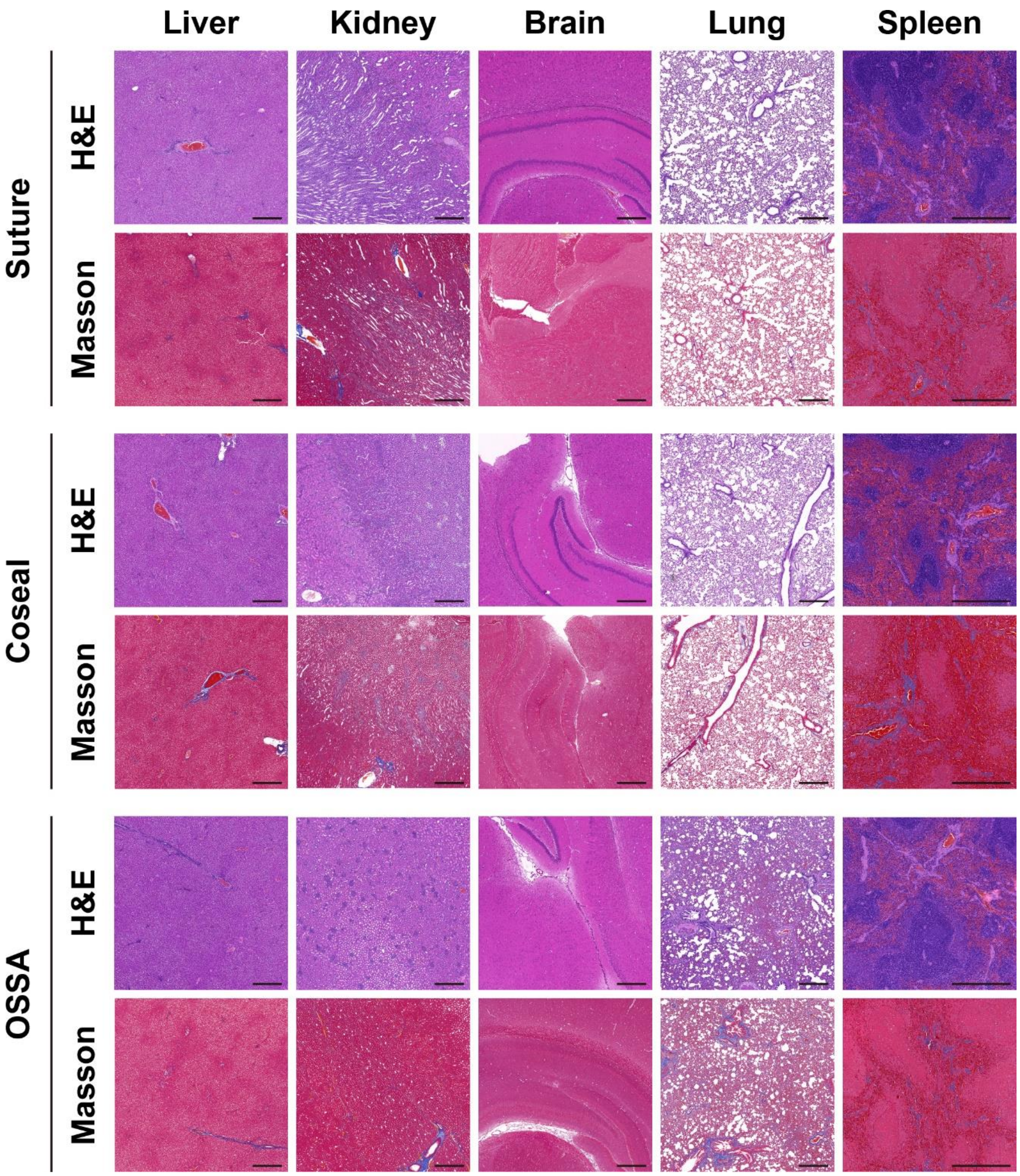

Supplementary Figure 20. H&E and Masson staining for livers, kidneys, brains, lungs, and spleens of the rats with gastric defects treated with the suture, Coseal, and OSSA bioadhesives, respectively (n=8 independent samples). Scale bar: 50 µm. Three areas per tissues were randomly selected and analyzed.

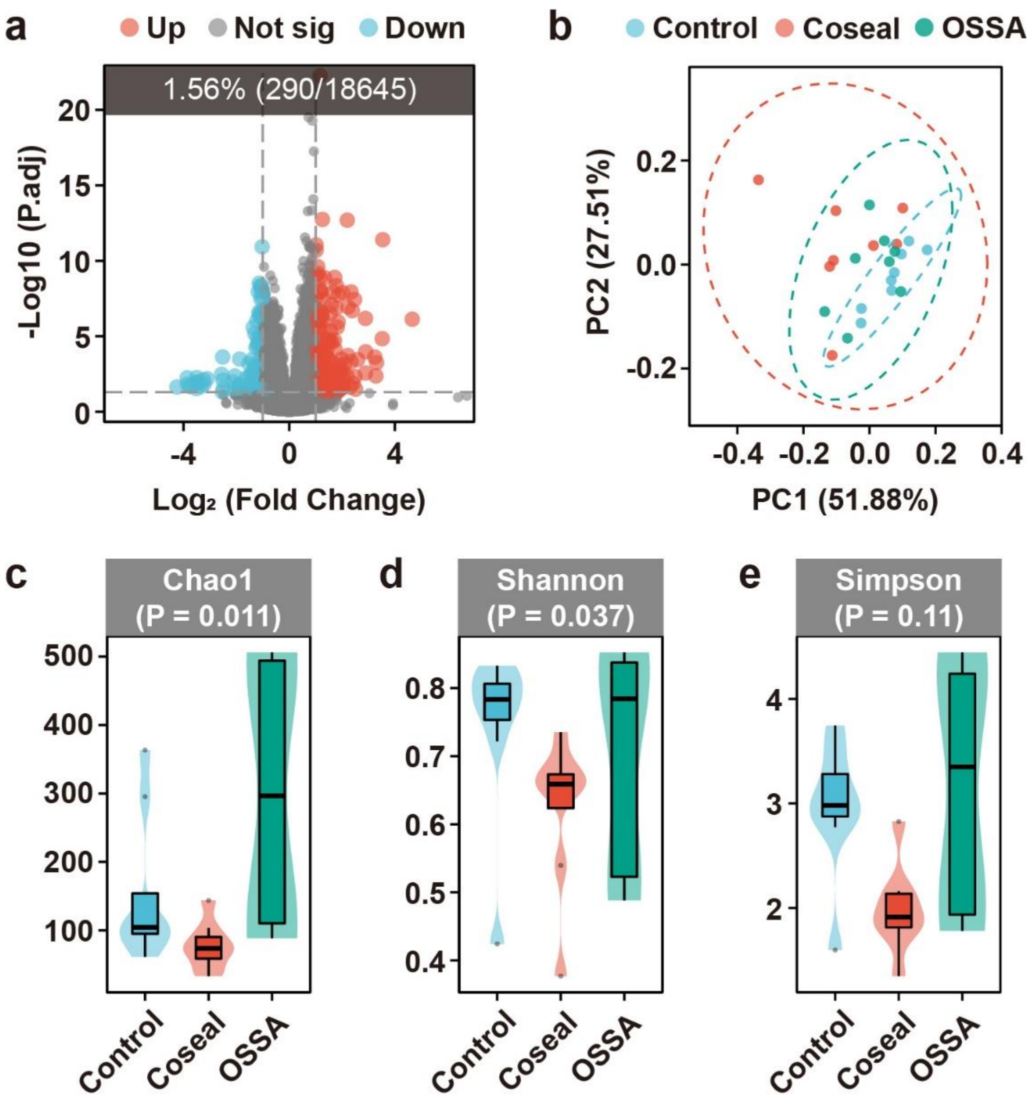

Supplementary Figure 21. Changes in transcriptome and microbiome after gastric defect repair. a) The volcano plot displaying the differentially expressed genes ($P < 0.05$, Fold Change > 1 or < -1) in Coseal group compared to the control group. The percentage and number of the differentially expressed genes in all detected genes were indicated in white. **b**) PCoA analysis reflecting the similarities in transcriptomes of stomach epithelium of the rats in the control, Coseal and OSSA groups. **c-e**) The α diversities analysis by Chao1, Shannon, and Simpson methods comparing the homogeneity of gastric microbiota.

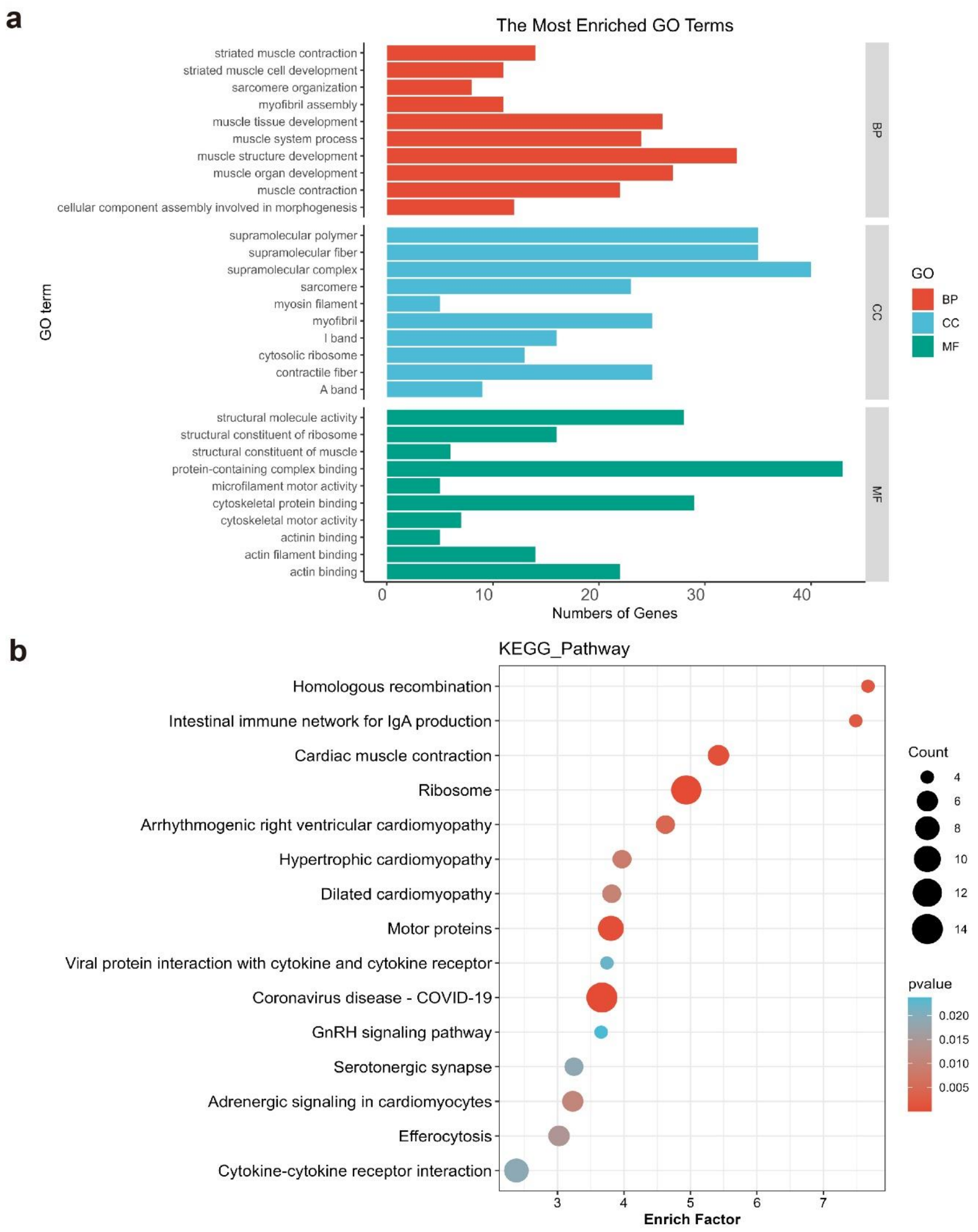

Supplementary Figure 22. The potential molecular mechanisms of OSSA bioadhesive promoting gastric tissue regeneration in a rat gastric defect model. a) Enrichment results of differentially expressed genes between the OSSA bioadhesive and control group in molecular function, biological process, and cellular component. **b**) Enriched pathways of differentially expressed genes between the OSSA bioadhesive and the control group.

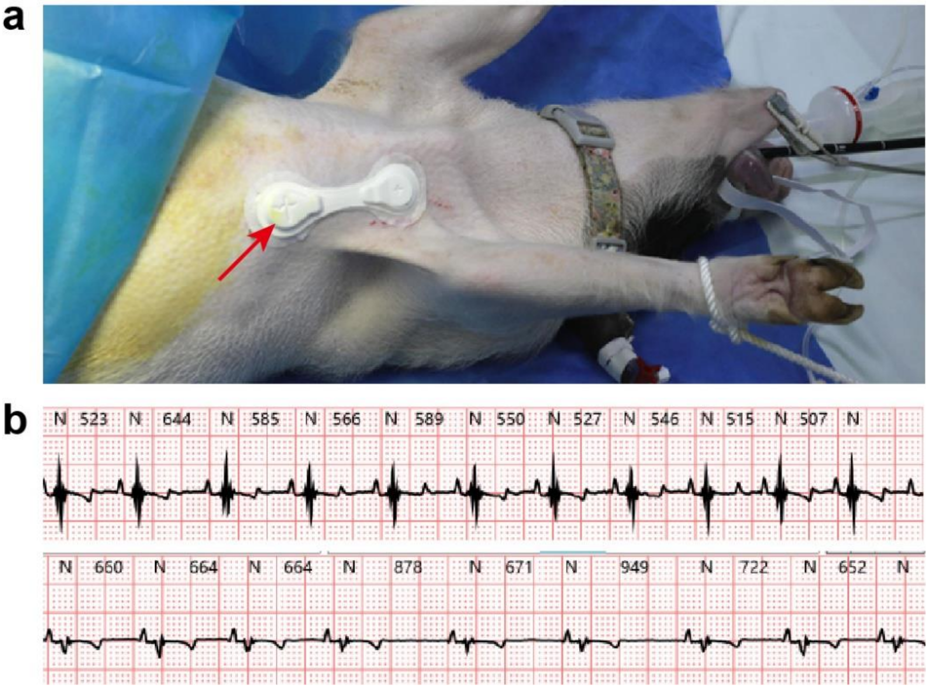

Supplementary Figure 23. Dynamic detection of electrocardiographic changes of porcine models. a) Representative image of electrocardiograph monitoring device for porcine models. **b**) Representative electrocardiographic results of porcine models.

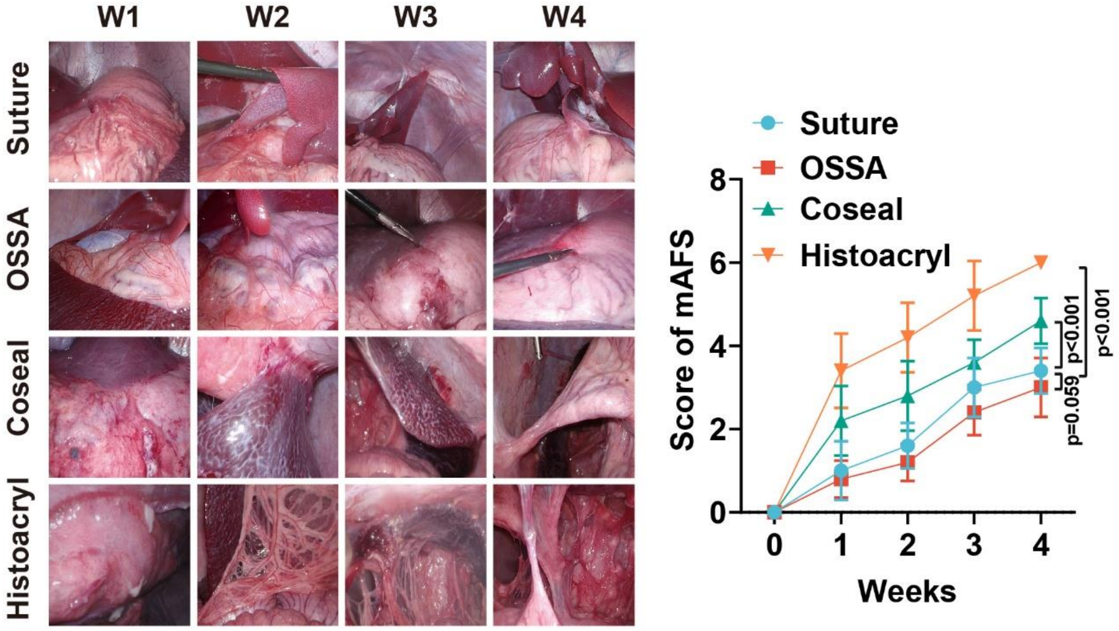

Supplementary Figure 24. The peritoneal adhesion conditions of porcine models with gastric defects after treatment with the suture, OSSA, Coseal, and Histoacryl adhesives. The representative images display the adhesion conditions with laparoscopic technique at 0, 1, 2, 3, 4 weeks, respectively. Data are presented as mean ± SEM (n=5 independent samples). The curves show the scores of mAFS to compare peritoneal adhesion severities. P values were determined by two-way ANOVA followed by Least Significant Difference post hoc test. Source data are provided as a Source Data file.

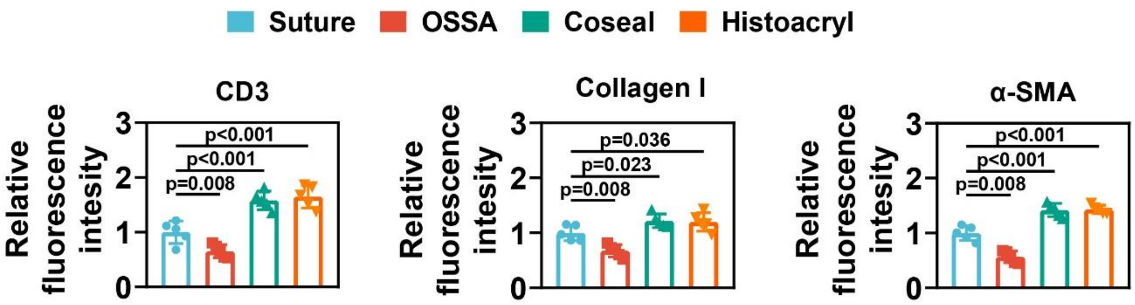

Supplementary Figure 25. The relative immunofluorescent densities of gastric tissues marked with CD3, Collagen I and α-SMA after sealing with the suture, OSSA, Coseal, and Histoacryl groups, respectively. Data are presented as mean ± SEM (n=8 independent samples). P values were determined by one-way ANOVA with Least Significant Difference post hoc test. Source data are provided as a Source Data file.

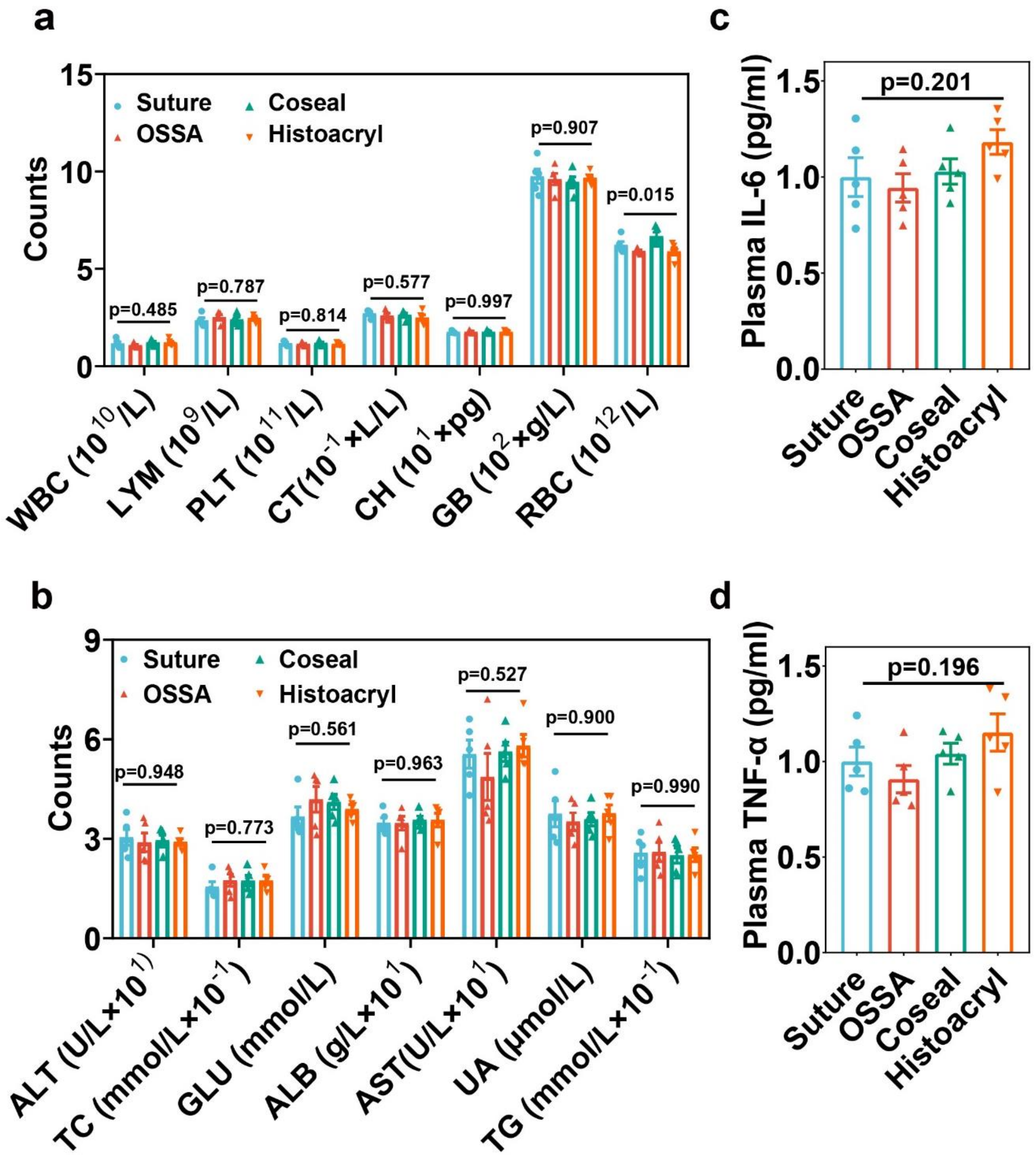

Supplementary Figure 26. *In vivo* biocompatibility of the suture, OSSA, Coseal, and Histoacryl adhesives using porcine models. a-d) Blood routine, biochemistry, IL-6, and TNF-α of the pigs with porcine gastric defects treated with the suture, OSSA, Coseal, and Histoacryl, respectively. Data are presented as mean ± SEM (n=5 independent samples). P values were determined by one-way ANOVA with Least Significant Difference post hoc test. Source data are provided as a Source Data file.
