# Supplementary material for: Acid-tolerant injectable bioadhesive for sutureless repair of large gastric perforation
Source: Nat Commun. 2026 Mar 24;17:4364. doi: 10.1038/s41467-026-71031-9 (PMC13172332; doi:10.1038/s41467-026-71031-9)
Supplement: Supplementary file 2 — Description of Additional Supplementary Files [file 41467_2026_71031_MOESM2_ESM.pdf]

Title: Supplementary Movie 1

Description: Water resistance measurement of OSSA adhesion to porcine skin.

Title: Supplementary Movie 2

Description: Sutureless repair of a porcine colon defect (diameter: 4 mm) after application and destruction of OSSA bioadhesive.

Title: Supplementary Movie 3

Description: Sutureless repair of a porcine stomach defect (diameter: 10 mm) by the OSSA bioadhesive.

Title: Supplementary Movie 4

Description: Leakage-free sealing of a porcine stomach defect (diameter: 20 mm) by the blue-stained OSSA bioadhesive.

Title: Supplementary Movie 5

Description: Sutureless repair of a gastric perforation (diameter: 6 cm) in an *ex vivo* porcine stomach by commercial Coseal adhesive.

Title: Supplementary Movie 6

Description: Sutureless repair of a gastric perforation (diameter: 6 cm) in an *ex vivo* porcine stomach by OSSA bioadhesive.

Title: Supplementary Movie 7

Description: Comparison of OSSA bioadhesive and surgical suture to seal gastric defects in rats.

Title: Supplementary Movie 8

Description: Establishment of the gastric perforation (diameter: 3 cm) in porcine models based on the combined laparoscopic-endoscopic technology.

Title: Supplementary Movie 9

Description: Endoscopic surveillance of the gastric defect model and 4-week repair efficiency in pigs.

Title: Supplementary Movie 10

Description: Laparoscopic surveillance of the gastric defect model and 4-week repair efficiency after laparoscopic delivery of injectable OSSA hydrogel in pigs.
