# Supplementary material for: Acid-tolerant injectable bioadhesive for sutureless repair of large gastric perforation
Source: Nat Commun. 2026 Mar 24;17:4364. doi: 10.1038/s41467-026-71031-9 (PMC13172332; doi:10.1038/s41467-026-71031-9)
Supplement: Supplementary file 13 — Reporting Summary [file 41467_2026_71031_MOESM13_ESM.pdf]

nature portfolio

Corresponding author(s): Xing Wang

Last updated by author(s): Feb 6, 2026

Reporting Summary

Nature Portfolio wishes to improve the reproducibility of the work that we publish. This form provides structure for consistency and transparency in reporting. For further information on Nature Portfolio policies, see our Editorial Policies and the Editorial Policy Checklist.

Statistics

For all statistical analyses, confirm that the following items are present in the figure legend, table legend, main text, or Methods section.

n/a	Confirmed	
[ ]	[x]	The exact sample size (n) for each experimental group/condition, given as a discrete number and unit of measurement
[ ]	[x]	A statement on whether measurements were taken from distinct samples or whether the same sample was measured repeatedly
[ ]	[x]	The statistical test(s) used AND whether they are one- or two-sided<br>*Only common tests should be described solely by name; describe more complex techniques in the Methods section.*
[x]	[ ]	A description of all covariates tested
[x]	[ ]	A description of any assumptions or corrections, such as tests of normality and adjustment for multiple comparisons
[ ]	[x]	A full description of the statistical parameters including central tendency (e.g. means) or other basic estimates (e.g. regression coefficient) AND variation (e.g. standard deviation) or associated estimates of uncertainty (e.g. confidence intervals)
[ ]	[x]	For null hypothesis testing, the test statistic (e.g. F, t, r) with confidence intervals, effect sizes, degrees of freedom and P value noted<br>*Give P values as exact values whenever suitable.*
[x]	[ ]	For Bayesian analysis, information on the choice of priors and Markov chain Monte Carlo settings
[x]	[ ]	For hierarchical and complex designs, identification of the appropriate level for tests and full reporting of outcomes
[x]	[ ]	Estimates of effect sizes (e.g. Cohen's d, Pearson's r), indicating how they were calculated

Our web collection on statistics for biologists contains articles on many of the points above.

Software and code

Policy information about availability of computer code

Data collection	None
Data analysis	SPSS v25.0

For manuscripts utilizing custom algorithms or software that are central to the research but not yet described in published literature, software must be made available to editors and reviewers. We strongly encourage code deposition in a community repository (e.g. GitHub). See the Nature Portfolio guidelines for submitting code & software for further information.

Data

Policy information about availability of data

All manuscripts must include a data availability statement. This statement should provide the following information, where applicable:

- Accession codes, unique identifiers, or web links for publicly available datasets
- A description of any restrictions on data availability
- For clinical datasets or third party data, please ensure that the statement adheres to our policy

The authors declare that the data supporting the conclusions of this study are available within the article and its Supplementary Information file or from the corresponding author upon reasonable request.

Research involving human participants, their data, or biological material

Policy information about studies with human participants or human data. See also policy information about sex, gender (identity/presentation), and sexual orientation and race, ethnicity and racism.

Reporting on sex and gender	This study did not address sex/gender differences
Reporting on race, ethnicity, or other socially relevant groupings	This study did not analyze data by race, ethnicity, or other socially relevant groupings.
Population characteristics	See above
Recruitment	This study did not involve recruitment
Ethics oversight	Department of General Surgery, the First Medical Center, Chinese PLA General Hospital, Beijing 100853, China

Note that full information on the approval of the study protocol must also be provided in the manuscript.

Field-specific reporting

Please select the one below that is the best fit for your research. If you are not sure, read the appropriate sections before making your selection.

[x] Life sciences [ ] Behavioural & social sciences [ ] Ecological, evolutionary & environmental sciences

For a reference copy of the document with all sections, see nature.com/documents/nr-reporting-summary-flat.pdf

Life sciences study design

All studies must disclose on these points even when the disclosure is negative.

Sample size	The sample sizes were determined based on relevant reported studies and experimental experiences.
Data exclusions	There are no data exclusions in this study.
Replication	The experimental indicators were examined or determined with triplicates.
Randomization	The animals were randomly grouped based on random number generation by the computer
Blinding	Random number generation by the computer was used for group allocation, so the results are not free from the potential effects of subjective thoughts of researchers. The other experiments were conducted based on protocols and instruments and they are also free from the potential effects of subjective thoughts.

Reporting for specific materials, systems and methods

We require information from authors about some types of materials, experimental systems and methods used in many studies. Here, indicate whether each material, system or method listed is relevant to your study. If you are not sure if a list item applies to your research, read the appropriate section before selecting a response.

Materials & experimental systems

n/a	Involved in the study	
[x]	[ ]	Antibodies
[ ]	[x]	Eukaryotic cell lines
[x]	[ ]	Palaeontology and archaeology
[ ]	[x]	Animals and other organisms
[x]	[ ]	Clinical data
[x]	[ ]	Dual use research of concern
[x]	[ ]	Plants

Methods

n/a	Involved in the study	
[x]	[ ]	ChIP-seq
[ ]	[x]	Flow cytometry
[x]	[ ]	MRI-based neuroimaging

Eukaryotic cell lines

Policy information about cell lines and Sex and Gender in Research

Cell line source(s)	American Type Culture Collection
Authentication	Short tandem repeat was used for authentication of used cell lines.

Mycoplasma contamination	All cell lines have been tested negative for mycoplasma contamination.
Commonly misidentified lines (See ICLAC register)	None.

Animals and other research organisms

Policy information about studies involving animals; ARRIVE guidelines recommended for reporting animal research, and Sex and Gender in Research

Laboratory animals	Male Sprague-Dawley (SD) rats (250-270 g, 8 weeks) and Bama miniature pigs (20-22 kg, 10 months)
Wild animals	None
Reporting on sex	We used male SD rats and Pama pigs for experiments in order to avoid the influences of sex on experimental results.
Field-collected samples	The environment kept 18-22 °C, 40%-70% relative humidity and 12/12 h light period. They were given with the sterile feed and water ad arbitrium. The rats and pigs were acclimatized to the feeding condition without any experimental intervention for 1 week to avoid the unpredictable influencing factors before purchase.
Ethics oversight	Ethics Committee of Chinese PLA General Hospital

Note that full information on the approval of the study protocol must also be provided in the manuscript.

Plants

Seed stocks	None
Novel plant genotypes	None
Authentication	None

Flow Cytometry

Plots

Confirm that:

- [x] The axis labels state the marker and fluorochrome used (e.g. CD4-FITC).
- [x] The axis scales are clearly visible. Include numbers along axes only for bottom left plot of group (a 'group' is an analysis of identical markers).
- [x] All plots are contour plots with outliers or pseudocolor plots.
- [x] A numerical value for number of cells or percentage (with statistics) is provided.

Methodology

Sample preparation	GES-1 cells were pretreated with DMEM, Coseal and OSSA hydrogel extracts for 24 h. The pretreated cells were collected and stained by AnnexinV-FITC/PI cell apoptosis kit.
Instrument	BD FACSymphony
Software	FlowJo
Cell population abundance	Our experiments are not involved in cell sorting by flow cytometry. GES-1 is a classical cell line, which has been already been authenticated by STR. Hence, its purity can be guaranteed. The densities and amount of harvested cells were calculated using the cell counter before flow cytometry.
Gating strategy	The gating strategy was determined based on clusters of live and dead cells according to the protocols of AnnexinV-FITC/PI cell apoptosis kit.

- [x] Tick this box to confirm that a figure exemplifying the gating strategy is provided in the Supplementary Information.
